# Large optical nonlinearity enhancement under electronic strong coupling

**DOI:** 10.1038/s41467-021-21739-7

**Published:** 2021-03-05

**Authors:** Kuidong Wang, Marcus Seidel, Kalaivanan Nagarajan, Thibault Chervy, Cyriaque Genet, Thomas Ebbesen

**Affiliations:** 1 ISIS & icFRC, University of Strasbourg and CNRS, 8 allée Gaspard Monge, Strasbourg, France; 2 Institute of Quantum Electronics, ETH Zürich, Zürich, Switzerland

**Keywords:** Optics and photonics, Atomic and molecular physics

## Abstract

Nonlinear optical responses provide a powerful way to understand the microscopic interactions between laser fields and matter. They are critical for plenty of applications, such as in lasers, integrated photonic circuits, biosensing and medical tools. However, most materials exhibit weak optical nonlinearities or long response times when they interact with intense optical fields. Here, we strongly couple the exciton of cyanine dye J-aggregates to an optical mode of a Fabry-Perot (FP) cavity, and achieve an enhancement of the complex nonlinear refractive index by two orders of magnitude compared with that of the uncoupled condition. Moreover, the coupled system shows an ultrafast response of ~120 fs that we extract from optical cross-correlation measurements. The ultrafast and large enhancement of the optical nonlinar coefficients in this work paves the way for exploring strong coupling effects on various third-order nonlinear optical phenomena and for technological applications.

## Abstract (web summary)

Nonlinear optical response of the material plays a crucial role in light-matter interactions and is important for practical applications. Here, the authors report enhancement of optical nonlinearity of J-aggregate cyanine molecules due to strong coupling between the molecules and an optical cavity.

## Introduction

Third-order optical nonlinear effects are intrinsic characteristics of a material. Because many significant optical nonlinear phenomena, such as four wave mixing, optical modulation, self-focusing and stimulated Raman scattering, are caused by the third-order optical nonlinear susceptibility^1^, they have been explored extensively in various materials ranging from metals^2^, semiconductors^3–5^, 2D materials^6,7^, topological insulators^8^ to organic materials^9–13^. An ideal nonlinear optical material should possess large refractive index change at low optical power. In addition, a short response time is also crucial for photonics and optoelectronics applications^7,14^. Usually, the third-order optical nonlinear responses of a material can be described by two parameters, the nonlinear refractive index *n*_2_ and nonlinear absorption coefficient *β*. These nonlinear coefficients are related to the change in refractive index ∆*n* and the modification in attenuation coefficient ∆*α* of the material by *n*_2_ = Δ*n*/*I* and *β* = *Δα/I* when two-photon absorption dominates the nonlinear absorption processes, where *I* is the peak intensity of the optical beam^1^. Therefore, one of the central aims in nonlinear optics is to search for or design materials with large *n*_2_ or *β*. However, standard materials usually show weak nonlinear optical responses even under illumination with strong optical fields. Such limitations therefore call for alternative strategies in order to improve the nonlinear responses of existing materials.

**Fig. 1:**
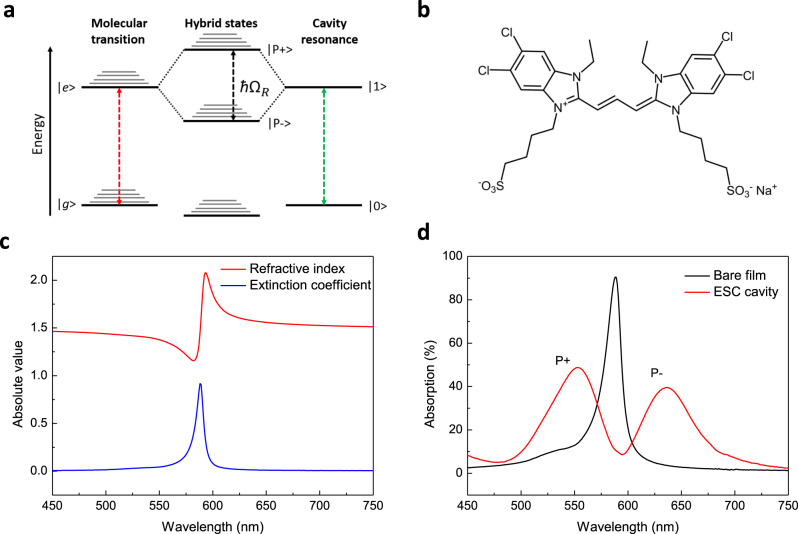
Light-matter strong coupling with organic semiconductors and linear responses of the samples. **a** Schematic energy diagram of the strong coupling between a molecular exciton transition and a cavity resonance. **b** Chemical structure of the J-aggregating cyanine monomer used in this work that eventually aggregate in the J-form. (See Methods). **c** Linear refractive index (red curve) and extinction coefficient (blue curve) of the bare J-aggregate molecular film calculated by transfer-matrix method. **d** Linear absorption spectra of the bare J-aggregate molecular film (no cavity - black curve) and of the molecular film under strong coupling (inside the FP cavity - red curve) at normal incidence.

One such strategy is to exploit the effects of light-matter strong coupling on materials’ optical responses. This can be achieved by coupling an excitonic transition and a resonant optical mode of a cavity and when the energy exchange between them is faster than the timescales associated with all dissipative and incoherent processes, two new exciton-polaritonic states are generated, separated in energy by the so-called Rabi splitting (Fig. 1a). Theory shows that such polaritonic states are generated even in the dark due to coupling with vacuum fluctuations of the cavity mode. It has been seen in the past years that the mere presence of such polaritonic states in the coupled system lead to new material properties. For instance, strongly coupled organic molecules could enhance the conductivity^15,16^, the rate of energy transfer^17^ of molecules, and furthermore, modify the work function^18^ and chemical reactions of molecules^19,20^. Besides, recent studies also showed that second-harmonic generation and third harmonic generation could be enhanced in the presence of polaritonic states^21–24^. However, the measurements in these works did not characterize the intrinsic nonlinear optical parameters such as *n*_2_ and *β*, which are necessary to evaluate the true potential of strong coupling for all nonlinear optical processes.

In this article, we applied Z-scan technique^25^ to characterize the nonlinear refractive index and nonlinear absorption coefficient of J-aggregate cyanine molecules that are placed either inside a Fabry-Perot (FP) cavity in electronic strong coupling (ESC) condition or outside of it (decoupled situation). As we show below, the formation of the hybrid light-matter states under ESC conditions gives rise to an enhancement of both *n*_2_ and *β* values larger than two orders of magnitude. Simulations and modeling show that the large improvement of the optical nonlinear coefficients results not only from the increase of the intracavity electric field at the polaritonic wavelengths, but also, and most remarkably, from an enhancement of the polaritonic dispersion third-order susceptibility itself. In addition, a pulse-width limited ultrafast response (~120 fs) of the coupled system is observed by means of optical pump-probe measurements. This result demonstrates how ESC can also meet the essential requirements for ultrafast optical modulation and data processing.

## Results

### Linear optical measurements

Our strongly coupled system (ESC cavity) was realized by placing J-aggregates of cyanine molecules dispersed in a polyvinyl alcohol (PVA) polymer inside a planar silver FP cavity. This type of molecule is selected essentially because of its narrow absorption linewidth^26^ (63 meV in our study) and its large nonlinear optical response^11^. The structure of the molecular monomer is shown in Fig. 1b and all details regarding samples’ preparation are given in Methods part. As a reference, we use a sample where the same organic material is spin coated on one mirror only, with therefore no possibility of strong coupling. From the linear absorption spectrum (1-T-R) of the bare molecular film, where the center wavelength of the exciton appears at 590 nm (Fig. 1d), we estimate the linear refractive index and extinction coefficient by transfer-matrix method^27^, as indicated in Fig. 1c. The molecular film exhibits a sharp peak in extinction coefficient, which corresponds to a distortion in the linear refractive index of PVA polymer according to Kramers-Krӧnig relation. Inside the cavity, when the absorption of the molecules is resonant with the optical mode of the cavity, the coupled system as expected, yields two exciton-polaritonic states (noted as |P + > and |P− > ) at wavelengths of 552 nm and 636 nm, with a Rabi splitting energy of 297 meV. The experimental and simulated spectra can be seen in Fig. 1d and Supplementary Fig. 1.

### Nonlinear optical measurements

**Fig. 2:**
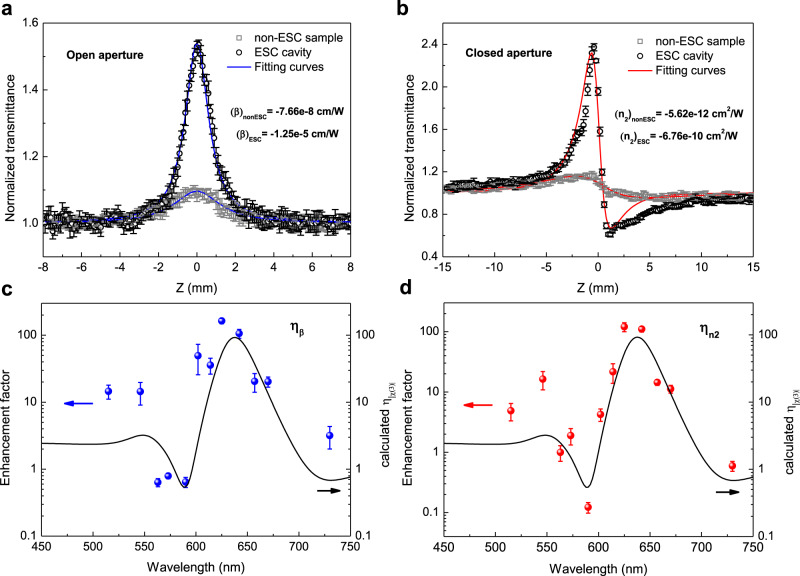
Nonlinear responses of the coupled and uncoupled systems. Open- **a** and closed-aperture **b** Z-scan traces of the J-aggregate cyanine molecules inside (open black circles) and outside (open gray squares) the FP cavities at 625-nm optical illumination. The focal intensity for the non-ESC sample and ESC cavity are 54.5 and 2.73 GW/cm^2^, respectively. The fitting curves in both (**a** and **b**) are obtained with a saturation model that is discussed in Methods part. **c**,**d** Are enhancement factors of the retrieved *n*_2_ (blue circles) and *β* (red circles) for various wavelengths. The black curves in (**c** and **d**) are the calculated enhancement factor of the absolute values of third-order susceptibility from a simplified nonlinear Lorentz model discussed below. The error bars of each point are calculated from at least three sets of repeated data.

We investigated the open- and closed-aperture Z-scan measurements in visible range to characterize the nonlinear optical response of the ESC cavity and non-ESC sample. The details of the setup can be found in Supplementary Note 2. The open- and closed-aperture Z-scan traces were recorded for different peak intensities and confirm the nonlinear nature of the optical response of the samples near the focus of the beam. As illustrated in Supplementary Note 3, the difference between the peak and the valley of the normalized transmittance (∆*T*_p−v_) increases nearly linearly with the peak intensity for both the ESC cavity and non-ESC sample at the representative wavelength (640 nm). This indicates that the optical response in Z-scan measurements is caused by a third-order optical nonlinear process^25^. It should be noted here that the molecules are more easily damaged at wavelengths near resonance inside the cavity than when placed outside the cavity. Therefore, lower peak intensities were applied for the Z-scan measurements in the ESC cavity compared with that in the non-cavity sample. The Z-scan traces are directly related to the nonlinear refractive index and the nonlinear absorption coefficient. The values of *β* can be retrieved from the profiles of open-aperture Z-scan with a saturation model that described in Methods part, whereas when analyzing *n*_2_ from the closed-aperture Z-scan traces, the nonlinear absorption should also take into account because the energy variations near the center of the transmitted beam stem from both *n*_2_ induced extra phase front distortion of the beam and the absorption change of the sample near the focus^25^. The transmissive Z-scan traces of the molecules inside and outside the cavities for both the open and closed apertures under the irradiance of 625 nm light are displayed in Fig. 2a, b, respectively. The parameters used for the laser, the measured saturable intensity of the J-aggregate molecular film and the Z-scan traces of the open- and closed-aperture measurements at the different wavelengths are presented in Supplementary Notes 4 and 5, respectively.

This leads us to compare, between the ESC and non-ESC cases, the nonlinear coefficients, *n*_2_ and *β*, over the full visible range, and in this way to define the enhancement factors of both coefficients as $\eta _{n_2}$ = (*n*_2_)_ESC_/(*n*_2_)_nonESC_ and *η*_*β*_ = (*β*)_ESC_/(*β*)_nonESC_, where (…)_ESC_ and (…)_nonESC_ represent the absolute values of the nonlinear coefficients of the molecules inside and outside the ESC cavities, respectively, under the same illumination wavelength. As can be clearly seen with a log scale in Fig. 2c, d, both *n*_2_ and *β* are strongly enhanced under ESC compared to the non-ESC sample, with a trend that remarkably follows the linear absorption spectrum of the coupled system inside the ESC cavity. In particular, $\eta _{n_2}$ and *η*_*β*_ reach the maximum values of 120 and 163 near the wavelength of the lower polaritonic state, 625 nm. These two-orders-of-magnitude enhancements result in the values of −6.76 × 10^−10^ cm^2^/W and −1.25 × 10^−5^ cm/W for *n*_2_ and *β*, respectively, which are more than one order of magnitude larger than those of engineered plasmonic metamaterial^28^, and higher than those at recently reported for J-aggregate cyanine molecules^11^ and that of the nonlinear indium tin oxide in the epsilon-near-zero region^4^. In contrast, enhancement factors <1 are measured in between the two polaritonic peaks, i.e., at the middle of Rabi splitting at 590 nm where only dark collective polaritonic states exist that cannot couple with the incident light. A reduction of the nonlinear coefficients also arises in the spectral regions far from the polaritonic peaks where most of the incident light at this wavelength is reflected by the front silver film (see Supplementary Fig. 1). Besides, the measured values of *β* for both the strongly coupled and uncoupled systems have different signs depending on the optical wavelength. The negative and positive values of *β* indicate saturable absorption and reverse saturable absorption, respectively^11^, as detailed in Supplementary Note 5.

**Fig. 3:**
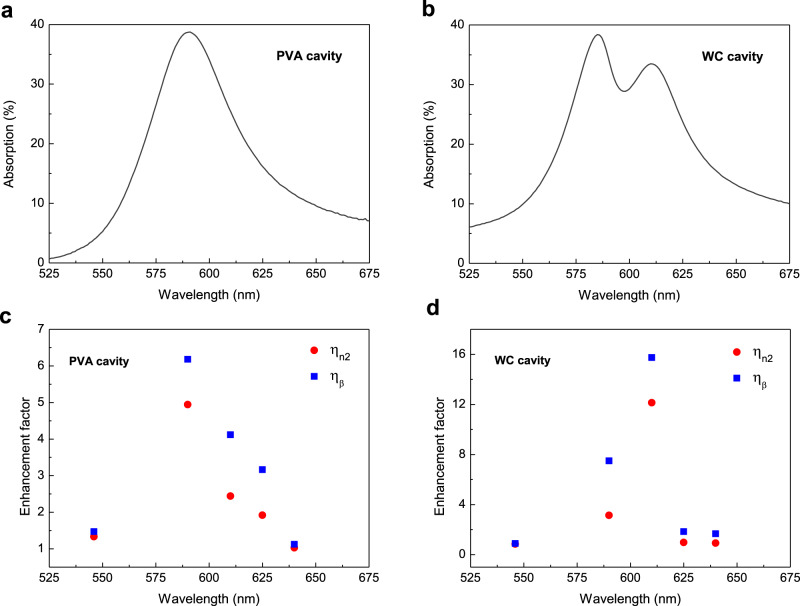
Linear and nonlinear responses of the PVA cavity and weak-coupling system. Linear absorption spectrum of (**a**) PVA cavity and (**b**) WC cavity. **c**, **d** Are enhancement factors of *n*_2_ (red circles) and *β* (blue squares) for PVA and WC cavities, respectively, at several representive wavelengths.

In order to discriminate and understand the influence of pure cavity resonance on such nonlinear enhancements shown above, we carried out wavelength-dependent Z-scan measurements on a pure PVA film, either placed inside and outside the cavities (uncoupled system). Due to the dispersive nature of the cavity-induced nonlinear response, the thickness of the PVA film was precisely selected to be the same as that of the molecule-doped PVA film inside the ESC cavity. The linear absorption spectrum shows that the resonant wavelength of this cavity appears at 590 nm, as shown in Fig. 3a. By comparing the nonlinear coefficients of the PVA cavity and the non-cavity sample, the dispersive enhancement of *n*_2_ and *β* is measured and given in Fig. 3c. Here, $\eta _{n_2}$ and *η*_*β*_ possess maximum values of 4.9 and 6.2 at the resonant wavelength (590 nm) for the uncoupled system, values that are much smaller than those measured for the strongly coupled system. This indicates that the cavity effect just plays a minor role in the large optical nonlinear enhancements near the polaritonic wavelengths under strong coupling condition. In addition, the optical nonlinear response of the excitonic resonance also needs to be explored because the exciton effect can potentially enhance the nonlinear response of some organic materials^29^. For this purpose, we investigated Z-scan measurements on a cavity-exciton weak-coupling (WC) system that does not support polaritons. To meet the requirement of the WC condition, the concentration of the cyanine molecules was reduced to a range where the coupling strength is smaller than the homogeneous decay rates of the cavity and the excitonic resonances^30^. A small dip in the cavity resonance in linear absorption spectrum can be seen in Fig. 3b. In our experiment, the Z-scan measurements were performed at multiple wavelengths in order to precisely probe the dispersive nonlinear coefficients of the WC and non-cavity configurations. As presented in Fig. 3d, the enhancement of *n*_2_ and *β* for WC cavity are maximum at the wavelength of two peaks, reaching values of 12.1 and 15.8, respectively, at one of the exciton-cavity coupled states (610 nm). However, these WC-induced nonlinear enhancements are still much smaller than the polariton-induced values shown above.

**Fig. 4:**
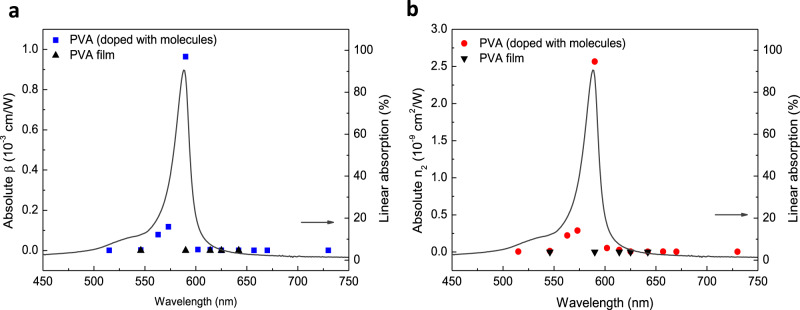
Nonlinear coefficients of PVA film with and without molecule doping. Absolute values of (**a**) nonlinear absorption coefficient and (**b**) nonlinear refractive index of the PVA film with (blue squares and red circles) and without molecules (black triangles), respectively. The gray solid curves represent the linear absorption spectrum of the molecules.

In parallel, the dispersive nonlinear coefficients of excitons that do not couple to any cavity mode can also be studied by measuring the Z-scan traces of the PVA film with and without molecule doping. As shown in Fig. 4, contrasting with the featureless dispersive nonlinearity of the pure PVA film, both *n*_2_ and *β* of the PVA doped with molecules possess large values at the excitonic wavelength, whereas they yield very small values at wavelengths far from the excitonic resonance. At wavelengths of 545 and 625 nm that correspond to the two polaritonic wavelengths in our strongly coupled system (see Fig. 1d), the nonlinear coefficients of the molecules are comparable with those of pure PVA film. This implies that the exciton itself plays only a minor role in the measured optical nonlinearity enhancement at the polaritonic wavelengths inside the ESC cavity. Therefore, the comparisons above clearly indicate that the polaritonic states dominate the enhancement in both nonlinear optical coefficients under strong coupling condition.

**Fig. 5:**
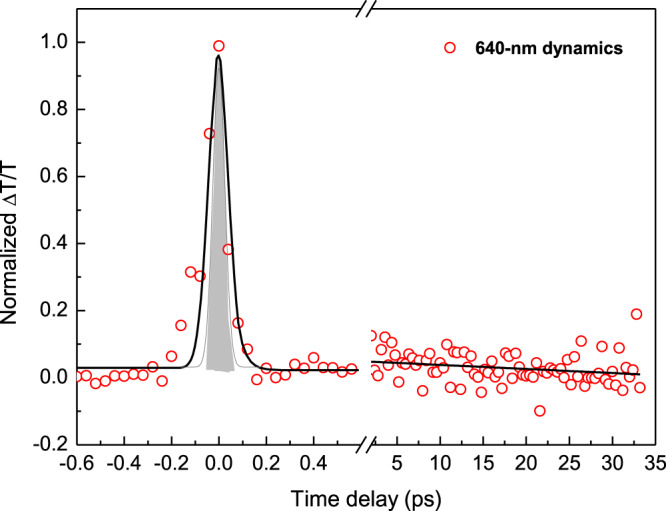
Ultrafast response of the coupled system. Normalized ultrafast transmittance of the ESC system under a 640 nm optical illumination. The gray area shows the pulse intensity profile (in arbitrary unit).

The temporal response of the molecules under strong coupling was also explored with a degenerate pump-probe measurement at 640 nm. Here, the amplitude of the transmitted probe light is modulated by illuminating a pump beam, with the pulse width of both beams measured to be 59 fs. The result displayed in Fig. 5 shows an ~120-fs (full width half maximum) peak at time zero, followed by a weak signal with an exponential decay over tens of picoseconds. The zero-delay peak originates in the optical Kerr effect and has a duration proportional to the convolution of the temporal envelope of the probe pulse and the temporal response function of the pump-induced dynamic process in the coupled system. The duration of this Kerr nonlinearity can be even smaller when the pulse width of the pump and probe pulses reduces, indicating it is an upper limit of the intrinsic response time of the strongly coupled system. The temporal response of the slow picosecond component is related to the lifetime of the cyanine molecules^31^ and its modulation amplitude is much smaller than that of the Kerr type zero-delay peak. The femtosecond time response of the coupled system here is shorter than that of optical nonlinear semiconductors^4,32,33^, and is thus ideal for ultrafast optical switching applications.

As indicated by the results presented above, we attribute the large enhancement values of the nonlinear optical coefficients under strong coupling mainly to the enhancement of the electric field intensity inside the cavity and to the polariton-assisted dispersive third-order susceptibility.

**Fig. 6:**
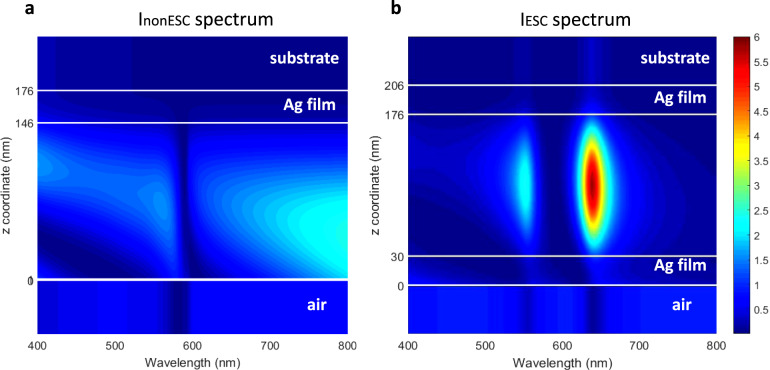
Electric field intensity of the samples. **a,b** Electric field intensity spectra of molecules outside (**a**) and inside (**b**) the FP cavities along *z* axis.

To see this, we first compare the electric field intensity distributions inside the cavity under ESC (*I*_ESC_) and outside the cavity -the non-ESC sample (*I*_nonESC_) - along the optical *z* axis. This leads us defining an enhancement factor for the electric field intensity as *η*_*I*_ = *I*_ESC_/*I*_nonESC_. According to the mean field approximation presented in^34,35^, one can evaluate the enhancement factor of the third-order susceptibility (*χ*^(3)^) of the molecules as

1 $$\eta _{\chi 3} = f\frac{{\left\langle {\left| {{\mathbf{E}}_{{\mathrm{ESC}}}} \right|^2} \right\rangle _V\left\langle {{\mathbf{E}}_{{\mathrm{ESC}}}^2} \right\rangle _V}}{{\left\langle {\left| {{\mathbf{E}}_{{\mathrm{nonESC}}}} \right|^2} \right\rangle _V\left\langle {{\mathbf{E}}_{{\mathrm{nonESC}}}^2} \right\rangle _V}}$$

where **E**_ESC_ and **E**_nonESC_ are the electric field distribution within the molecular layer inside and outside the cavities, respectively, and <…>_*V*_ denotes an average of the field intensities taken inside the film over a given volume enclosing a volume fraction *f* of molecules. Since our molecular film is homogeneous (and composed of only one type of optically active molecules) we fix *f* = 1. Assuming $\left\langle {\left| {{\mathbf{E}}_{{\mathrm{\delta}}}} \right|^2} \right\rangle _V\left\langle {{\mathbf{E}}_{{\mathrm{\delta}}}^2} \right\rangle _V \sim I_{{\mathrm{\delta}}}^2$ (δ = ESC, non ESC), the enhancement factor of $\chi ^{(3)}$ is directly related to the enhancement factor for the electric field intensity with $\eta _{\chi 3} = \eta _I^2$. This assumption is reasonable because the complex dielectric constant is dominated by its real part, especially for wavelengths near to the wavelengths of the polaritonic states. The field intensity enhancement factor can be directly evaluated from the simulations shown in Fig. 6^36^. For our experimental conditions, the comparison between the results of Fig. 6a, b gives a maximal intensity enhancement $\eta _I$ distribution along *z* axis at the lower polariton wavelength. Consider the electric field intensity at 636 nm, the beam size at focal point is much larger than the thickness of the film, thereby the transverse electric field can be regarded as unchanged throughout the pumped unit volume. For the longitudinal electric field intensity, the $I_{{\mathrm{ESC}}}$ near the middle of the film is ~6, which is much larger than the values of 1 at the two edges, i.e., $\eta _I$ at the central part of the molecular volume dominates the enhancement of nonlinear susceptibility. Therefore, when taking into account the $I_{{\mathrm{nonESC}}}$ value at 636 nm (1.5), $\eta _I$ is calculated to be ~4 at the lower polaritonic wavelength and hence the corresponding $\eta _{\chi 3}$ is ~16. Furthermore, from the optical Kerr effect by a single beam, intensity-dependent complex nonlinear refractive index $\tilde n_2$ can be described by^1,37^

2 $$\tilde n_2 = \frac{3}{{4n_0n_0^\prime \varepsilon _0c}}\chi ^{(3)}$$

where $\tilde n_2 = n_2 + in_2^{\prime\prime} = n_2 + i\frac{c}{{2\omega }}\beta$, here $n_0$ and $n_0^{\prime}$ are the complex and the real part of the linear refractive index, respectively. $n^{\prime\prime}_2$ is the imaginary part of the nonlinear refractive index, $\varepsilon _0$ and $c$ are the permittivity and the light velocity in vacuum. This indicates that $\tilde n_2$ can be enhanced with the same magnitude as $\chi ^{(3)}$ when the electric field intensity is boosted under strong coupling condition. Accordingly, as evaluated above, the enhancement factors of $n_2$ and $\beta$ are 16 at the lower polaritonic wavelength. In addition, the enhancement of the electric field intensity at the wavelength of lower polariton is obviously larger than that at the upper polaritonic wavelength, which is consistent with the enhancement spectra of both $n_2$ and $\beta$ in Fig. 2c, d, a trend that confirms that $\eta _I$ contributes to the enhancement of nonlinear coefficients. But remarkably, these 16-times enhancement values remain smaller than the two-orders-of-magnitude enhancement on nonlinear coefficients measured experimentally (Fig. 2c, d). This difference points toward other causes for the observed optical nonlinear enhancement under strong coupling.

**Fig. 7:**
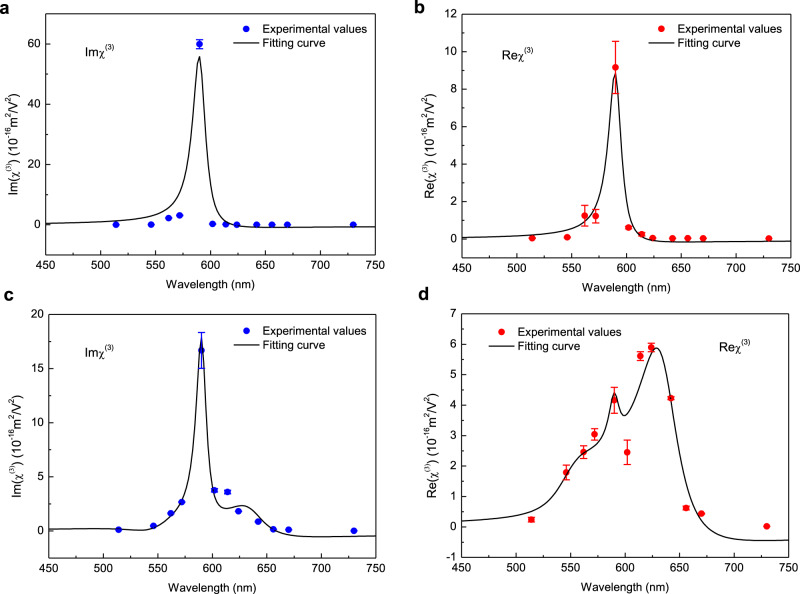
Experimental and fitted nonlinear susceptibilities of the coupled and uncoupled systems. Experimental and fitted (black curves) absolute values of the third-order susceptibility of the uncoupled (**a**, **b**) and strongly coupled (**c**, **d**) systems. The blue circles in (**a**, **c**) and red circles in (**b**, **d**) represent the imaginary and real parts of the nonlinear susceptibility. The black curves are obtained with the nonlinear Lorentz model given by Eq. (3), and the error bars of each point are calculated from at least three sets of repeated data.

As already stressed above, the data gathered in Fig. 2c, d reveal the signatures of clear resonance enhancements when the frequencies are close to the upper (UP) and lower (LP) polaritonic state energies. In addition, the results in Figs. 3 and 4 exclude (i) that exciton and cavity effects provide the main contribution to such resonance enhancements, and (ii) that weak-coupling conditions could be sufficient in reaching such nonlinear enhancements values. Taken together, these data clearly show that the polaritonic states are responsible for these enhancements. From this, it is then possible to follow a simple nonlinear Lorentzian model for the nonlinear third-order susceptibility involving the two polaritonic states and an excitonic state in the strongly coupled system. This model allows us to write^38^

3 $$\chi _{{\mathrm{ESC}}}^{\left( 3 \right)}(\omega ) = \mathop {\sum }\limits_{k = {\mathrm{UP}},{\mathrm{LP}},{\mathrm{ex}}} \left( {\frac{{\omega _k^2}}{{\omega _k^2 - \omega ^2 - i\gamma _k\omega }}} \right)\bar \chi _k^{(3)}$$

where $\omega _k$ and $\gamma _k$ correspond to the frequency and linewidth of upper polariton ($k = {\mathrm{UP}}$), lower polariton ($k = {\mathrm{LP}}$) and exciton ($k = {\mathrm{ex}}$), respectively. $\omega$ is the frequency of the pump beam and $\bar \chi _k^{(3)}$ is the static third-order susceptibilities associated with each excited states involved. Before analyze the dispersive $\chi _{{\mathrm{ESC}}}^{\left( 3 \right)}$ of the strongly coupled system, we should first estimate the value of $\bar \chi _{{\mathrm{ex}}}^{(3)}$ of the excitonic state from the uncoupled system. For the uncoupled system, only the excitonic resonance contributes to the dispersive nonlinear susceptibility. Since the experimental values of the real and imaginary parts of the third-order nonlinear susceptibility can be estimated from the Z-scan results above based on Eq. (2), $\bar \chi _{{\mathrm{ex}}}^{(3)}$ is obtained to be $\left( { - 0.528-2.091{\mathrm{i}}} \right) \times 10^{ - 17}\,{\mathrm{m}}^2/{\mathrm{V}}^2$ by best fitting $\chi _{{\mathrm{nonESC}}}^{(3)}$, as presented in Fig. 7a, b. Then, using the measured values of $\omega _{{\mathrm{UP}}}$ (2.25 eV), $\gamma _{{\mathrm{UP}}}$ (0.201 eV), $\omega _{{\mathrm{LP}}}$ (1.95 eV) and $\gamma _{{\mathrm{LP}}}$ (0.141 eV) from the linear absorption spectrum of the coupled system (Fig. 1d), and fitting the best $\chi _k^{(3)}$ values with $\bar \chi _{{\mathrm{UP}}}^{\left( 3 \right)} = (0.747 - 0.842{\mathrm{i}}) \times 10^{ - 17}{\mathrm{m}}^2/{\mathrm{V}}^2$ and $\bar \chi _{{\mathrm{LP}}}^{(3)} = - (2.562 + 3.615{\mathrm{i}}) \times 10^{ - 17}{\mathrm{m}}^2/{\mathrm{V}}^2$, the dispersive nonlinear susceptibility of the coupled system can be well fitted, as illustrated in Fig. 7c, d. Furthermore, comparing the calculated dispersive nonlinear susceptibility of the strongly coupled and uncoupled systems, the enhancement of $\left| {\chi ^{\left( 3 \right)}} \right|$ directly yields the spectral dispersive features observed in enhancement of the nonlinear coefficients, as presented with black solid curves in Fig. 2c, d. This indicates that the polariton-induced dispersive $\chi _{{\mathrm{ESC}}}^{\left( 3 \right)}$ also contributes to the enhancement of the nonlinear optical coefficients. The details of the model can be found in Supplementary Note 6. A microscopic description of the underlying mechanisms is beyond the scope of this work. But we can speculate that, beside the resonance enhancement effect of the dispersive nature of the polaritonic states, the rather high values for the static third-order susceptibilities could be related to the large transition moments associated with the delocalized nature of the polaritonic states, in a similar way as in our previous work on polaritonic states- driven enhanced second-harmonic generation^21^.

## Discussion

Since both the large electric field intensity inside the cavity at the polaritonic wavelengths and the energy dispersion of $\chi _{{\mathrm{ESC}}}^{\left( 3 \right)}$ at the upper and lower polaritons contribute to the significant increase in the optical nonlinear coefficients, the enhancement factor of $n_2$ and $\beta$ can be improved if $(\eta _I)_{{\mathrm{UP}},{\mathrm{LP}}}$ and $(\chi _{{\mathrm{ESC}}}^{\left( 3 \right)})_{{\mathrm{UP}},{\mathrm{LP}}}$ are further increased. From the calculations, the $(\eta _I)_{{\mathrm{UP}},{\mathrm{LP}}}$ and $(\chi _{{\mathrm{ESC}}}^{\left( 3 \right)})_{{\mathrm{UP}},{\mathrm{LP}}}$ are very sensitive to the quality factor (QF) of the cavity, i.e., strongly increasing with QF. Considering the fact that the QF of the FP cavity is only 12 at 590 nm resonance in our experiments, there is much room to further enhance $(\eta _I)_{{\mathrm{UP}},{\mathrm{LP}}}$ and $(\chi _{{\mathrm{ESC}}}^{\left( 3 \right)})_{{\mathrm{UP}},{\mathrm{LP}}}$. For instance, distributed Bragg reflectors^39,40^, which are usually used in polariton condensation studies, and various nanostructures^30^ provide possible candidates for high QF cavities. The study of the third-order optical nonlinearity enhancement by exciton-polaritons in this work opens the gates to explore the effect of strong coupling on a series of third-order optical nonlinear phenomena, such as wave mixing, optical modulation and stimulated Raman scattering. In addition, the femtosecond intrinsic response time of the coupled system makes it possible to realize ultrafast optical switching for future applications.

In summary, we have performed Z-scan measurements on J-aggregate cyanine molecules inside a FP cavity in the ESC regime, and measured an enhancement by two orders of magnitude on both the nonlinear refractive index $n_2$ and nonlinear absorption coefficient $\beta$ at the lower polaritonic state compared with measurements done outside the cavity. These large nonlinear enhancements are ascribed to both the increase of the electric field intensity inside the cavity and the polaritonic dispersion of the third-order susceptibility. In addition, we also demonstrated an ultrafast response of ~120 fs of the coupled system using cross-correlation measurements. Such ultrafast, large optical nonlinearities in the strongly coupled system presented here offer an efficient way to realize high speed active photonic and optoelectronic devices.

## Methods

### Sample preparation

Three groups of samples were prepared for our experiments. The first group on strong-coupling measurements includes two samples. The first sample with molecules enclosed in an optical cavity (ESC sample) that can be strongly coupled to the cavity mode, and the second one without cavity (non-ESC sample), hence only uncoupled molecules exist. For the ESC sample, a 30 nm silver film was sputtered on the quartz window, and an ~146 nm polymer layer (PVA), doped with J-aggregate cyanine molecules TDBC (5,6-Dichloro-2-[[5,6-dichloro-1-ethyl-3-(4-sulfobutyl)-benzimidazol-2-ylidene]-propenyl]-1-ethyl-3-(4-sulphobutyl)-benzimidazolium hydroxide, inner salt, sodium salt, Few Chemicals), was spin coated on top of the silver film. Then, another 30 nm silver film was sputtered on top of the polymer to form a FP cavity. The thickness of the polymer film was carefully selected so as to have the cavity resonance and the molecular absorption peak overlapping. For the non-ESC sample, the thickness of the bottom silver film and TDBC-PVA layer are the same with those of the ESC sample, but without the addition of the top silver film (no cavity formed). The TDBC-PVA solution was made by mixing equal amount of 0.5 wt% TDBC water solution and 5 wt% PVA water solution (molar weight 205000, Sigma-Aldrich). The second group on WC experiments also includes two samples. The PVA film doped with a lower concentration of TDBC (made by the mixed solution of equal amount of 0.05 wt% TDBC water solution and 5 wt% PVA water solution), was placed inside and outside the FP cavities to form the weakly coupled and the non-cavity samples. The third group of samples related to non-coupled measurements were made by PVA film without TDBC doping: one sample with a PVA film inside the FP cavity, and the other without the cavity. The thickness of the Ag film and polymer film for the second and third groups of samples are identical to those for the samples in the first group, and the substrates for all the samples are 1 mm thick quartz windows.

### Estimation of optical nonlinear coefficients from Z-scan measurements

The extraction of the nonlinear absorption coefficient $\beta$ and nonlinear refractive index $n_2$ is only based here on the open- and closed-aperture transmissive Z-scan traces because the normalized changes in the reflective Z-scan are much smaller than that in the transmitted one for most of the cases, as displayed in Supplementary Fig. 4. In the case of the open aperture, the energy variations within the whole beam profile of the transmitted light were measured. Therefore, modifications in the transmittance only relate to the nonlinear absorption of the sample, being strongest at the focus and smallest far from it. A positive (negative) peak in the normalized transmittance near the focus corresponds to a negative (positive) value of $\beta$, which indicates reverse saturable absorption (saturable absorption). In the closed-aperture case, only the energy near the center of the transmitted beam is detected. Thus, variations in transmission are associated to both the nonlinear absorption and nonlinear-refraction induced self-focusing and defocusing. A valley-peak signal in closed-aperture Z-scan indicates a positive nonlinear refractive index and a peak-valley profile corresponds to a negative value of $n_2$.

Assuming a circular profile to the incident beam, the normalized transmittance in the Z-scan measurements can be expressed by

4 $$T = (1 - \alpha L)/(1 - \alpha _0L)$$

where $\alpha$ and $\alpha _0$ are the total and linear absorption coefficients, respectively. $L$ is the sample thickness. The linear absorption coefficient $\alpha _0$ at different wavelengths can be determined via $\alpha _0 = 4\pi n^{{\prime}}_0\kappa /\lambda$ when the linear refractive index ($n^{{\prime}}_0$) and the extinction coefficient ($\kappa$) are obtained from the measured absorption spectra.

Considering that both the saturation absorption and two-photon absorption contribute to the absorption response in Z-scan measurements, we have^41,42^

5 $$\alpha \left( I \right) = \frac{{\alpha _0}}{{1 + I_{\mathrm{e}}/I_{\mathrm{s}}}} + \beta I_{\mathrm{e}}$$

where $I_{\mathrm{e}}$ and $I_{\mathrm{s}}$ are excitation intensity and saturable intensity, respectively. $I_{\mathrm{e}}$ is related to the optical intensity at the focus $I_{\mathrm{p}}$, the Rayleigh length $z_0$ of the beam and the sample position $z$ by $I_{\mathrm{e}}\left( z \right) = I_{\mathrm{p}}/(1 + z^2/z_0^2)$. According to the expressions above, the normalized transmittance for open aperture can be given by

6 $$T_{{\mathrm{OA}}} = \left[ {1 - \frac{{\alpha _0I_{\mathrm{s}}L}}{{I_{\mathrm{s}} + I_{\mathrm{p}}/(1 + z^2/z_0^2)}} - \frac{{\beta I_{\mathrm{p}}L}}{{1 + z^2/z_0^2}}} \right]/(1 - \alpha _0L)$$

For closed-aperture Z-scan^25^,

7 $$T_{{\mathrm{CA}}} = \left( {1 - \frac{{4\Delta {{\Phi }}z/z_0}}{{\left( {z^2/z_0^2 + 9} \right)\left( {z^2/z_0^2 + 1} \right)}}} \right)$$

where $\Delta {{\Phi }} = 2\pi n_2I_{\mathrm{p}}L_{{\mathrm{eff}}}/\lambda$. $L_{{\mathrm{eff}}}$ is the effective length, which is associated with the linear absorption coefficient $\alpha _0$ by $L_{{\mathrm{eff}}} = \frac{{1 - e^{ - \alpha _0L}}}{{\alpha _0}}$. Therefore, the nonlinear coefficients $n_2$ and $\beta$ can be obtained by fitting the experimental Z-scan data with the equations above.

## Supplementary information

Supplementary Information

## Data Availability

The data that support the findings of this study are available from the corresponding authors upon reasonable request.
