# Supplementary material for: Large optical nonlinearity enhancement under electronic strong coupling
Source: Nat Commun. 2021 Mar 5;12:1486. doi: 10.1038/s41467-021-21739-7 (PMC7935967; doi:10.1038/s41467-021-21739-7)
Supplement: Supplementary file 1 — Supplementary Information [file 41467_2021_21739_MOESM1_ESM.pdf]

Supplementary Information for

Large optical nonlinearity enhancement under electronic strong coupling

Kuidong Wang[1], Marcus Seidel[1], Kalaivanan Nagarajan[1], Thibault Chervy[2], Cyriaque Genet[1*] & Thomas Ebbesen[1*]

[1]University of Strasbourg, CNRS, ISIS & icFRC, 8 allée Gaspard Monge, 67000 Strasbourg, France. [2]Institute of Quantum Electronics, ETH Zürich, CH-8093 Zürich, Switzerland.

Email: genet@unistra.fr, ebbesen@unistra.fr

Supplementary Note 1: Linear response of the strongly coupled system

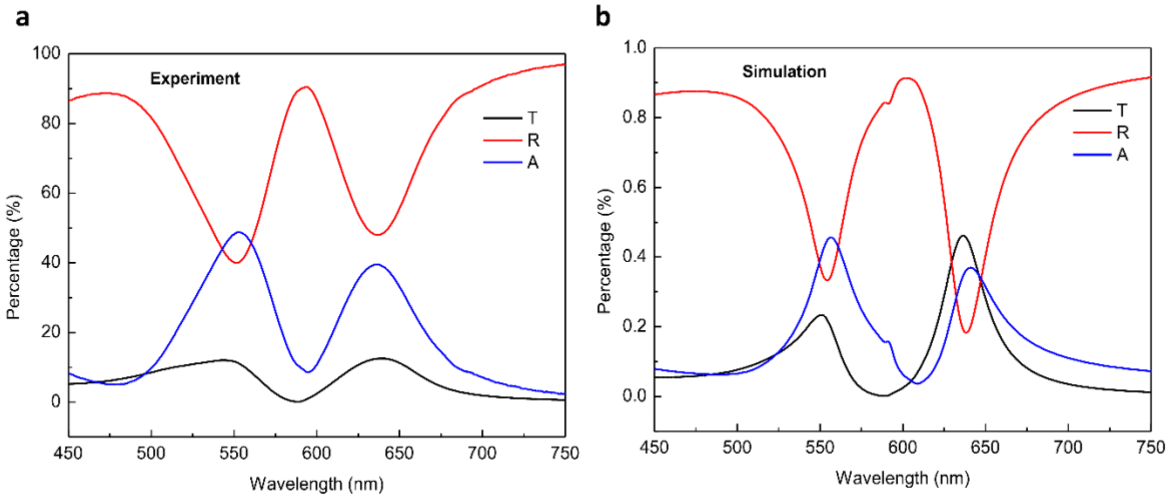

Supplementary Figure 1. Linear measurements of the strongly coupled system. **a** Measured and **b** calculated linear transmittance (black curves), reflectance (red curves) and absorption spectra (blue curves) of the strongly coupled system.

The linear transmittance, reflectance and absorption spectra of the ESC sample were measured at normal incidence using a commercial spectrophotometer (Model V-670, JASCO), as shown in Supplementary Fig. 1a. For all the spectra, two resonances are clearly observed, at wavelengths of 552 and 636 nm, which represent upper and lower polaritonic states. Similar features of the spectra were also obtained by transfer matrix simulation[1]. Here, the simulated amplitudes of the transmittance (Supplementary Fig. 1b) at the two resonances are slightly different from that of the measured values, a discrepancy that may stem from the imperfections of the cavity.

Supplementary Note 2: Z-scan setup

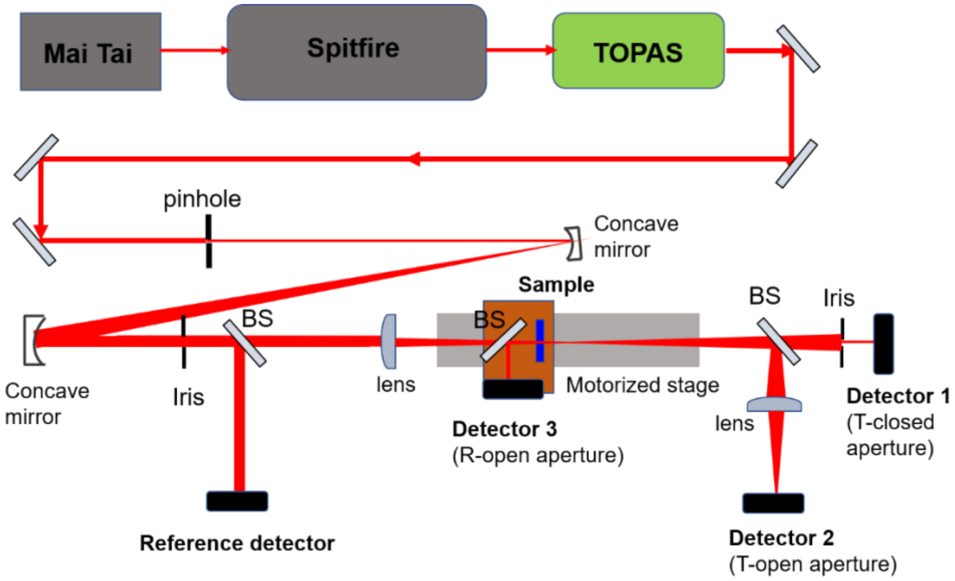

Supplementary Figure 2. Experimental setup. Schematics of transmissive and reflective Z-scan experimental setup.

A schematic of the setup used for Z-scan experiments is given in Supplementary Fig. 2. The tunable visible beams involved in our experiments are generated by an optical parametric amplifier

pumped with an 800-nm, 100-fs laser beam generated by a regenerative amplifier (Spitfire, Spectra-Physics). The output light was first passed through a 500-μm pinhole to form a circular symmetric Airy beam. Then, the beam diameter was increased and collimated by a pair of concave mirrors. Diffracted rings far from the optical axis of the beam were trimmed by an iris. This circular beam was finally guided to Z-scan setup. Here, the first beam splitter (BS) is used to route one part of the beam to a reference photodetector in order to monitor the energy fluctuations during the experiments. The second and third BSs are used to guide the reflected beam from the sample surface and split the transmitted beam into two portions for the open- and closed-aperture measurements, respectively. The second BS, detector 3 and the sample were mounted on a motorized translation stage. It should be noted that for the reflective Z-scan, we just investigated the open-aperture experiments because the size of the reflected beam unavoidable varies when the stage is moving.

Supplementary Note 3: Intensity-dependent open- and closed-aperture Z-scan traces for the ESC cavity and non-ESC sample

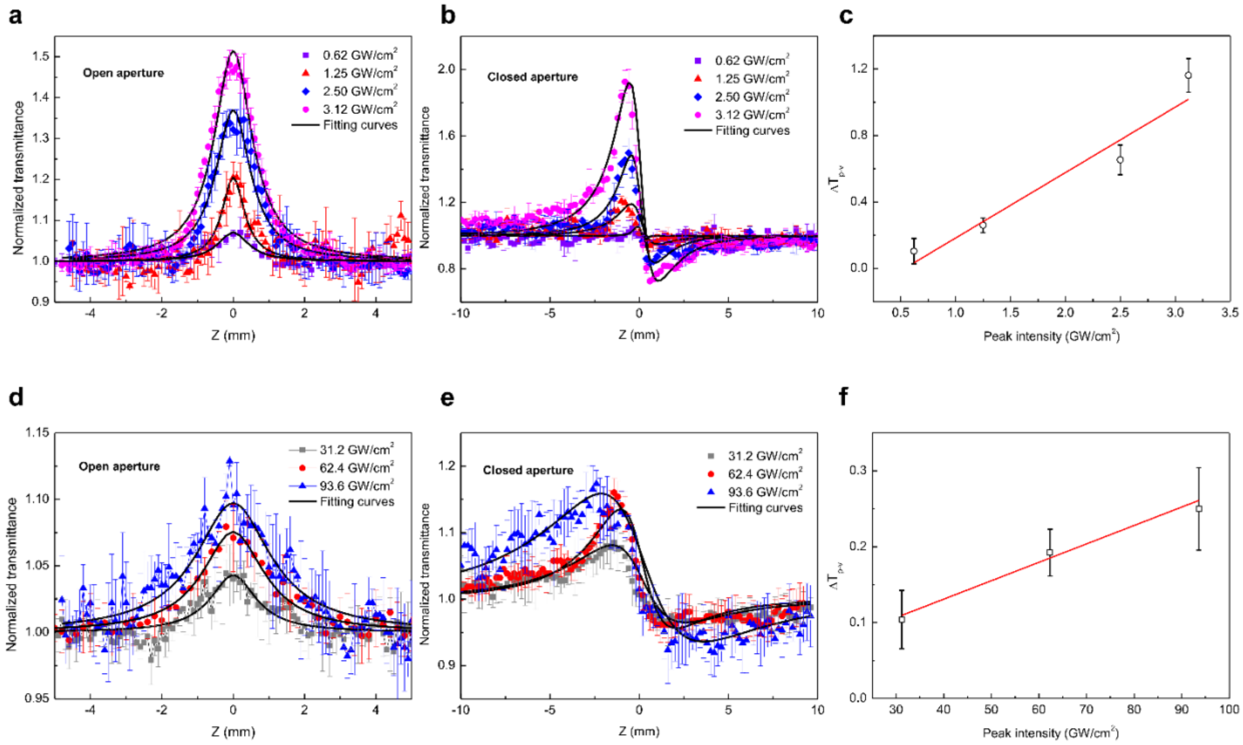

Supplementary Figure 3. Z-scan traces as a function of the radiated intensity. Open- **a, d** and closed- **b, e** aperture transmissive Z-scan traces of the ESC cavity (upper panel) and non-ESC sample (lower panel) acquired at various peak intensities under 640-nm light irradiance. **c** and **f** are extracted peak-intensity-dependent ΔT_{p-v} from **b** and **c**, respectively. Red curves are the fitting of the values of ΔT_{p-v}. The error bars of each point are calculated from at least 3 sets of repeated data acquisitions.

In order to confirm the nonlinear nature of the optical response of both the ESC cavity and non-ESC sample near the focal point, the open- and closed-aperture Z-scan traces were recorded at the various peak intensities (Supplementary Figs. 3a, 3b, 3d, 3e). Since the damage threshold of the samples is quite different for the molecules inside and outside the cavities, we applied different range of peak intensities here. As shown in Supplementary Figs. 3b and 3e, the peak-valley contrast

are more pronounced while the peak intensity increases. The differences between the peak and valley of the normalized transmittance ($\Delta T_{\mathrm{p-v}}$) increase nearly linearly with the peak intensity for both the ESC cavity and non-ESC sample (Supplementary Figs. 3c, 3f). This indicates that the optical response in Z-scan measurements is caused by a third-order optical nonlinear process[2].

Supplementary Note 4: Parameters of the optical beam and molecular film at various wavelengths

Supplementary Table 1. Parameters of the optical beam and the saturation intensity of the molecular film at different wavelengths.

Wavelength (nm)	Beam size at focal point (μm)	Pulse width (fs)	Saturation intensity (GW/cm^2)
515	15.5	88.0	51.3
546	14.2	84.0	28.9
563	14.7	78.0	6.00
573	16.2	68.0	2.99
590	19.5	76.0	0.959
602	18.4	70.0	10.1
614	18.5	62.6	31.1
625	18.9	65.4	109
640	19.1	59.0	124
657	19.2	56.9	124
670	18.4	68.7	149
730	16.3	68.0	106

The beam size at the focal point for the different wavelengths listed in Supplementary Table 1 were estimated from fitting the Z-scan traces with the saturation model (see Methods part). The

pulse widths were measured with a commercial autocorrelator (Delta Basic Model + UV options, Minioptic Technology, Inc.). The saturable intensities of the bare TDBC-PVA film were determined by measuring transmittance at various peak intensities.

Supplementary Note 5: Z-scan results at various wavelengths

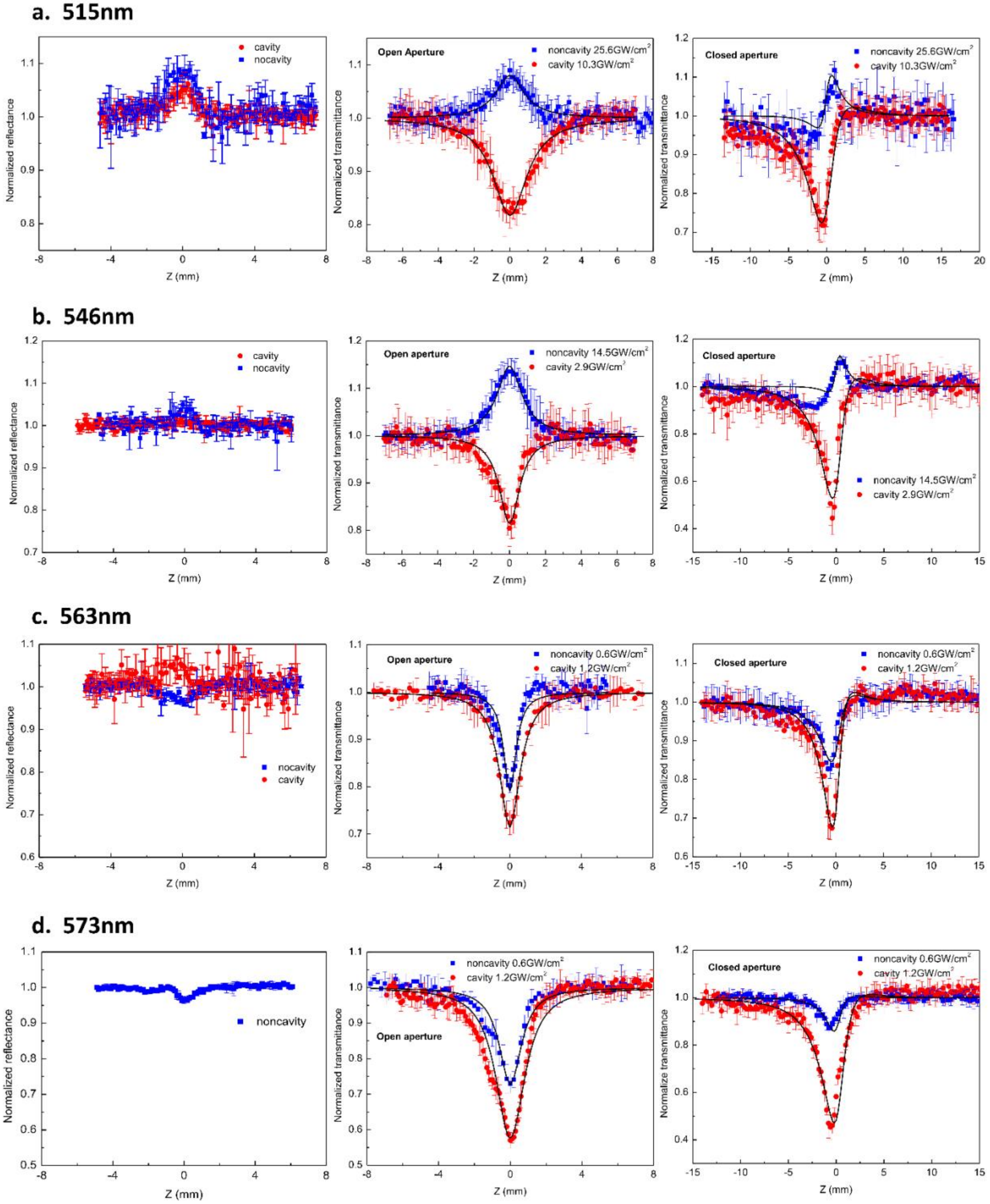

e. 590nm

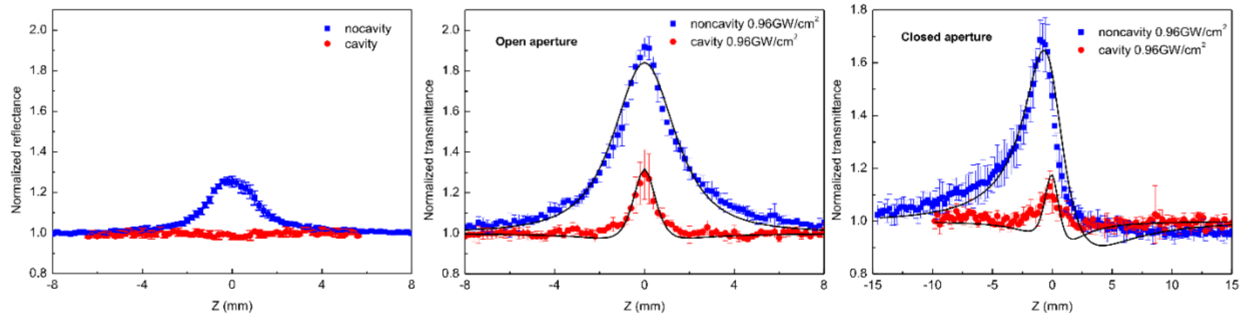

f. 602nm

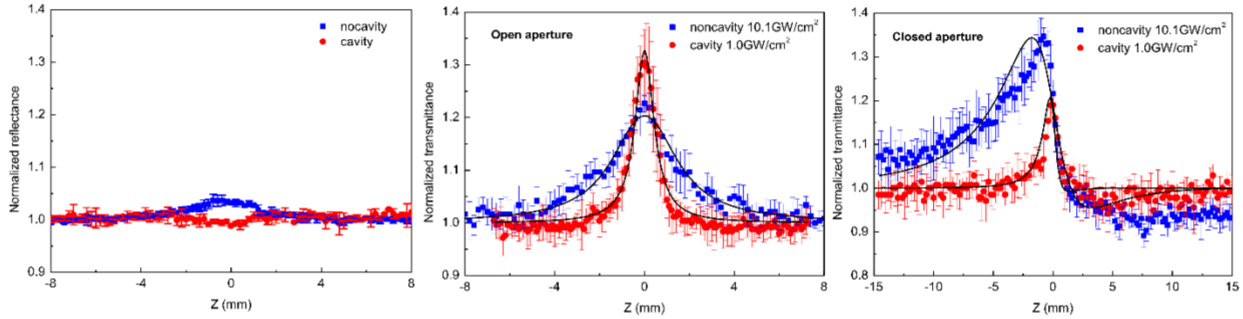

g. 614nm

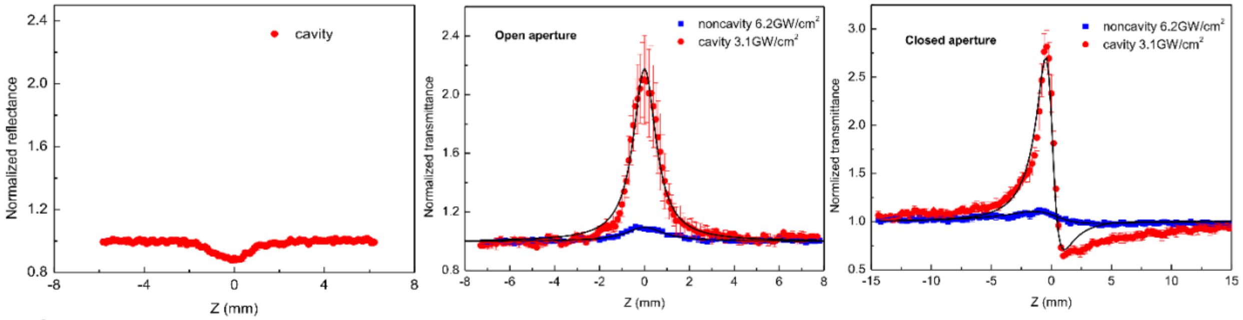

h. 625nm

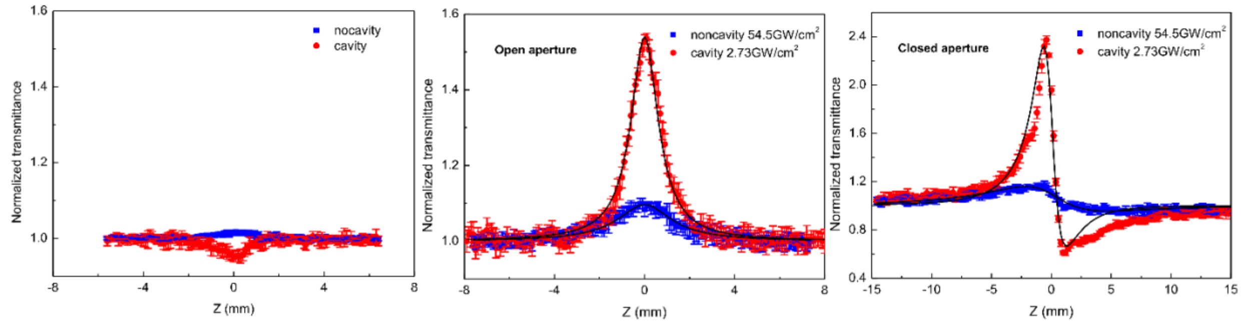

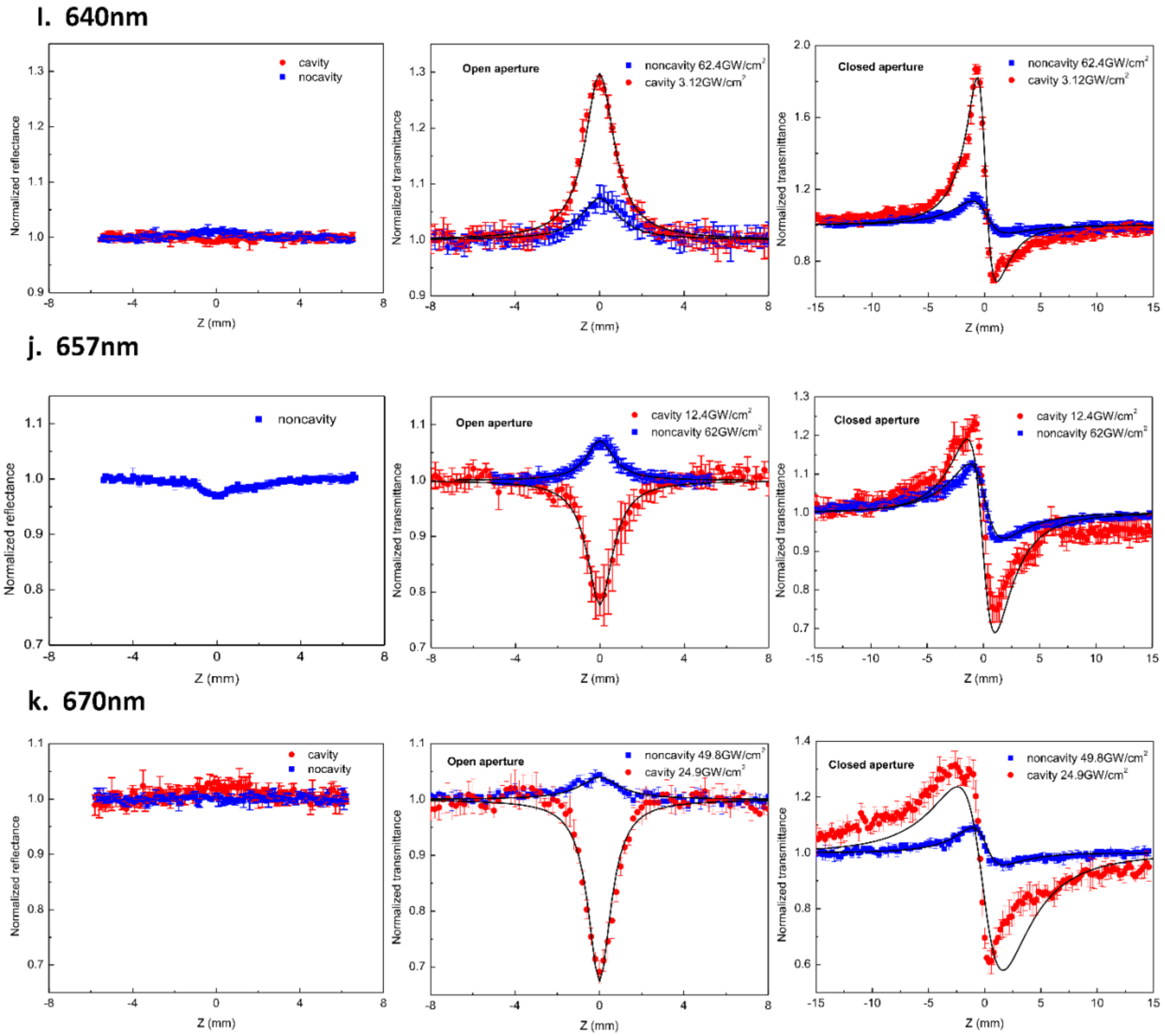

Supplementary Figure 4. Z-scan traces at various wavelengths. Open- and closed-aperture Z-scan traces recorded at **a** 515 nm, **b** 546 nm, **c** 563 nm, **d** 573 nm, **e** 590 nm, **f** 602 nm, **g** 614 nm, **h** 625 nm, **I** 640 nm, **j** 657 nm and **k** 670 nm. For each figure, the left, middle and right panels show the results of open-aperture reflective Z-scan, open- and closed-aperture transmissive Z-scan, respectively. The red closed circles and blue squares in all figures represent the Z-scan data acquired from ESC and non-ESC samples, respectively. The black fitting curves are obtained with a saturation model, and the error bars of each point are calculated from at least 3 sets of repeated data acquisitions.

The traces of the open- and closed-aperture transmissive Z-scan and the open-aperture reflective Z-scan at various wavelengths are displayed in Supplementary Fig. 4. Obviously, the normalized changes in the reflective Z-scan are much smaller than that in the transmitted one in most cases. Therefore, we mainly take into account the open- and closed-aperture transmissive Z-scan traces in our study when extracting the nonlinear absorption coefficient β and nonlinear refractive index n_2. As illustrated in Supplementary Fig. 4, different peak intensities were chosen here at the different optical wavelengths not only to avoid any damage of the samples under illumination, but also to achieve measurable variations in transmittance. For the results of the open-aperture Z-scan, the values of β are negative (positive) when the transmission is enhanced (suppressed) near the focus. As shown in the middle panel of each figures in Supplementary Fig. 4, the molecules exhibit different signs of β when changing the pump wavelengths, those positive values of β correspond to a typical two-photon absorption process and the negative values of β come from saturation absorption. For the closed-aperture Z-scan traces, a valley-peak signal indicates a positive nonlinear refractive index and a peak-valley profile corresponds to a negative value of n_2. It should be noted here that, during the extraction of the nonlinear coefficients of the ESC and non-ESC samples, we do not take into account the nonlinear effects of the quartz substrates and the silver films, because they yield very small values for n_2 and β [3, 4], and the variations in their transmittance cannot be resolved with our illumination maximum intensity.

Supplementary Note 6: Simplified model on third-order nonlinear susceptibility

In order to correlate the third-order nonlinear susceptibility and the formation of polaritonic states in strongly coupled systems, we consider a simplified nonlinear Lorentz model. In its simple formulation, the nonlinear Lorenz model consider the time evolution of the polarization density P of the system as damped harmonic oscillator with[5]

$$\frac{d^2P}{dt^2} + \gamma \frac{dP}{dt} + \omega_0^2 P = \omega_0^2 \varepsilon_0 \{\bar{\chi}^{(1)} E + \bar{\chi}^{(2)} E^2 + \bar{\chi}^{(3)} E^3 + \cdots\} \tag{S1}$$

where γ and ω_0 represent the linewidth and frequency of the transition, respectively. $\bar{\chi}^{(1)}$, $\bar{\chi}^{(2)}$ and $\bar{\chi}^{(3)}$ correspond to linear, second-order and third-order static susceptibilities, respectively. Using the Fourier transform and assuming that each transition is associated with an independent oscillator, we can get the third-order dispersion equation from the equation (S1) as

$$\chi^{(3)}(\omega) = \sum_k \left(\frac{\omega_k^2}{\omega_k^2 - \omega^2 - i\omega\gamma_k}\right) \bar{\chi}_k^{(3)} \tag{S2}$$

where ω_k, γ_k and $\bar{\chi}_k^{(3)}$ are the frequency, linewidth and third-order static susceptibility of the k oscillator, respectively. For the uncoupled system, where only excitons exist, we replace the subscript k by ex only, representing the parameters of the excitonic oscillator. Similarly, for the coupled system, we use UP and LP to describe the parameters associated with the upper and lower polaritonic oscillators.

Following this model, the dispersive susceptibility of the uncoupled and coupled systems studied in our experiments can be respectively expressed by

$$\chi_{\mathrm{nonESC}}^{(3)}(\omega) = \left(\frac{\omega_{\mathrm{ex}}^2}{\omega_{\mathrm{ex}}^2 - \omega^2 - i\omega\gamma_{\mathrm{ex}}}\right) \bar{\chi}_{\mathrm{ex}}^{(3)} \tag{S3}$$

$$\chi^{(3)}_{\mathrm{ESC}}(\omega) = \left(\frac{\omega^2_{\mathrm{UP}}}{\omega^2_{\mathrm{UP}} - \omega^2 - i\omega\gamma_{\mathrm{UP}}}\right)\bar{\chi}^{(3)}_{\mathrm{UP}} + \left(\frac{\omega^2_{\mathrm{LP}}}{\omega^2_{\mathrm{LP}} - \omega^2 - i\omega\gamma_{\mathrm{LP}}}\right)\bar{\chi}^{(3)}_{\mathrm{LP}} + \left(\frac{\omega^2_{\mathrm{ex}}}{\omega^2_{\mathrm{ex}} - \omega^2 - i\omega\gamma_{\mathrm{ex}}}\right)\bar{\chi}^{(3)}_{\mathrm{ex}} \quad \text{(S4)}$$

Based on equation (2) in the main text, we can calculate the values of both the complex nonlinear refractive index $\tilde{n}_2$ (Supplementary Table 2) and complex third-order nonlinear susceptibility (red and blue circles in Fig. 7 in the main text) from the results of the Z-scan measurements. For the uncoupled system, only the excitonic oscillator contributes to the dispersive nonlinear susceptibility. Here, we first decompose equation (S3) into real and imaginary parts, then use the linear values of $\omega_{\mathrm{ex}} = 2.1$ eV, $\gamma_{\mathrm{ex}} = 0.063$ eV (Fig. 1d in the main text) and fit the corresponding parts of $\chi^{(3)}_{\mathrm{nonESC}}$, as presented in Figs. 7a and 7b in the main text, in order to obtain the best fitting $\bar{\chi}^{(3)}_{\mathrm{ex}}$ value of $(-0.528 - 2.091\mathrm{i}) \times 10^{-17}\ \mathrm{m}^2/\mathrm{V}^2$. The acquisition of $\bar{\chi}^{(3)}_{\mathrm{ex}}$ allows us to further analyze the contribution of the polaritonic states to the dispersive nonlinear susceptibility of the coupled system. For the coupled system, the polaritonic parameters ω_{UP}, γ_{UP}, ω_{LP} and γ_{LP} can be extracted from the linear absorption spectrum (Fig. 1d in the main text) to be 2.25, 0.201, 1.95 and 0.141 eV, respectively. With these values, the real and imaginary parts of the dispersive susceptibilities of the coupled system can be well fitted, as shown in Figs. 7c and 7d in the main text, giving the fitting values for $\bar{\chi}^{(3)}_{\mathrm{UP}} = (0.747 - 0.842\mathrm{i}) \times 10^{-17}\mathrm{m}^2/\mathrm{V}^2$ and $\bar{\chi}^{(3)}_{\mathrm{LP}} = -(2.562 + 3.615\mathrm{i}) \times 10^{-17}\mathrm{m}^2/\mathrm{V}^2$. With this fitted nonlinear Lorenz model, we can eventually calculate the polariton-induced dispersive nonlinear susceptibility and the corresponding enhancement factor of the absolute values of $\chi^{(3)}$ ($\eta_{|\chi 3|}$) from the fitting curves in Fig. 7 in the main text, which can be found in Figs. 2c and 2d in the main text.

Supplementary Table 2. Extracted $\tilde{n}_2$ from the Z-scan measurements (unit: 10^{-16} m^2/W)

Wavelength (nm)	non-ESC sample	ESC sample
515	0.047-0.018i	0.24+0.11i
546	0.093-0.054i	1.79+0.47i
563	1.25+2.24i	2.46+1.63i
573	1.22+3.10i	3.04+2.67i
590	-9.16-59.9i	4.16-16.7i
602	-0.611-0.267i	-2.45-3.76i
614	-0.259-0.145i	-5.61-3.60i
625	-0.048-0.016i	-5.89-1.82i
640	-0.039-0.010i	-4.23-0.86i
657	-0.045-0.010i	-0.62+0.15i
670	-0.039-0.007i	-0.44+0.02i
730	-0.029-0.006i	-0.018+0.012i

Supplementary References

1. Katsidis CC, Siapkas DI. General transfer-matrix method for optical multilayer systems with coherent, partially coherent, and incoherent interference. *Applied optics* 2002, **41**(19)**:** 3978-3987.

2. Sheik-Bahae M, Said AA, Wei T-H, Hagan DJ, Van Stryland EW. Sensitive measurement of optical nonlinearities using a single beam. *IEEE journal of quantum electronics* 1990, **26**(4)**:** 760-769.

3. Boyd RW. *Nonlinear optics*. Elsevier, 2003.

4. Milam D. Review and assessment of measured values of the nonlinear refractive-index coefficient of fused silica. *Applied optics* 1998, **37**(3)**:** 546-550.

5. Varin C, Bart G, Fennel T, Brabec T. Nonlinear Lorentz model for the description of nonlinear optical dispersion in nanophotonics simulations. *Optical Materials Express* 2019, **9**(2)**:** 771-778.
